# Selection of In Vivo Relevant Dissolution Test Parameters for the Development of Cannabidiol Formulations with Enhanced Oral Bioavailability

**DOI:** 10.3390/pharmaceutics17010079

**Published:** 2025-01-09

**Authors:** Nathan Koch, Quentin Bourcy, Olivier Jennotte, Patrice Chiap, Anna Lechanteur, Jean-Michel Cardot, Brigitte Evrard

**Affiliations:** 1 Laboratory of Pharmaceutical Technology and Biopharmacy, Center for Interdisciplinary Research on Medicines (CIRM), University of Liège, 4000 Liège, Belgium; 2 Department of Toxicology, Center for Interdisciplinary Research on Medicines (CIRM), Academic Hospital of Liège, 4000 Liège, Belgium; 3 Borvo, 63211 Ceyrat, France

**Keywords:** Cannabidiol, in vitro–in vivo, dissolution test, biorelevant, bioavailability

## Abstract

**Background:** Cannabidiol (CBD) shows interesting therapeutic properties but has yet to demonstrate its full potential in clinical trials partly due to its low solubility in physiologic media. Two different formulations of CBD (amorphous and lipid-based) have been optimized and enable an increase in bioavailability in piglets. In vivo studies are time-consuming, costly and life-threatening. Therefore, we need to develop in vitro tests that can predict what will happen in vivo. **Methods:** Comparisons in terms of dissolution were made especially by using different media (FaSSGF, FaSSIF, FeSSIF, HCl 0.1N with or without SLS, phosphate buffer pH 6.8 with or without SLS) and different conditions (sink or non-sink conditions). These in vitro results were confronted with in vivo results to select the most appropriate dissolution test conditions. **Results:** The importance of the presence of surfactants to enable solubilization of CBD was demonstrated. Neutral media enabled a relatively good prediction of the extent of absorption observed in vivo, whereas the rate of absorption was more complicated to predict. **Conclusions:** FeSSIF media, and FaSSIF sink media to a lesser extent, were the only compositions enabling predictions of both extent and rate, indicating that emulsification is possibly a major contributor to the in vivo availability of the drug.

## 1. Introduction

The development of medicines containing active pharmaceutical ingredients (APIs) with a poor aqueous solubility is a major issue encountered by the pharmaceutical industry [1]. In total, 40% of the actual drugs and at least 70% of the new drug candidates face low bioavailability mainly due to the increase in lipophilicity that accompanies the discovery of new drug targets [2,3]. Cannabidiol (CBD) is a terpenophenolic compound classified as a BCS II drug, extracted from *Cannabis sativa* species belonging to this group of problematic drugs. In addition to its low aqueous solubility, CBD exhibits a good permeation but an important presystemic metabolism which impairs the blood concentrations and contributes to its low oral bioavailability [4]. This extensive first-pass metabolism participates not only in lowering the oral bioavailability but also contributes to the highly inter- and intra-patient variability of the pharmacokinetics of CBD [5,6]. Registered to reduce seizures in the context of Lennox-Gastaux and Dravet Syndrome (Epidiolex^®^) as an oral oily solution and to reduce spasticity induced by multiple sclerosis as an hydro-alcoholic solution in combination with 9Δ–tetrahydrocannabinol (Sativex^®^), CBD is also intensively studied and shows therapeutic effects, such as antioxidant and anti-inflammatory properties [7]. This molecule is also currently studied to treat psychotic disorders, drug abuse, insomnia, pain or anxiety [8]. As a lipophilic drug, CBD is often formulated with lipid excipients and taken with high-fat meal [9]. However, many other strategies have been investigated. For example, amorphous solid dispersions (ASDs) of CBD have been successfully developed as well as CBD-cyclodextrin complexes, lipid-based formulations, and mesoporous loaded silica [10]. These formulations have shown their ability to increase the CBD solubility in in vitro dissolution tests. Recently, significative enhancement of CBD oral bioavailability has been observed by giving an amorphous formulation, consisting of stabilized amorphous CBD within mesoporous silica, and a lipid mixture of Gelucire^®^ 50/13 and CBD, to fasted piglets [11]. These formulations complete the list of suitable strategies to increase the oral bioavailability of CBD recently reviewed by Hossein et al. [12]. For instance, a lipid-based formulation composed of self-assembly colloidal delivery systems called VESIsorb^®^ enabled a 4.4-fold increase in CBD plasma concentration in healthy humans, compared with CBD solubilized in medium chain triglycerides [13]. Other polymer-based strategies, such the use of a CBD–Poloxamer 407 preparation, and solid-based CBD preparations, like Arvisol^®^ (Echo Pharmaceuticals, Leiden, The Netherlands) composed of an oral tablet containing pure CBD, have shown an improvement in bioavailability [12]. The development and the optimization of such formulations imply the use of characterization tests to estimate the in vivo fate of the drug as accurately as possible. Many dissolution settings exist and the choice of a suitable medium, apparatus or hydrodynamic may be challenging [14]. Dissolution tests could range from very simple uses, such as the classic use of equipment and compendial media, to complex systems involving the combination of several pieces of equipment and the use of complex media. It is important to develop a test that is as simple and inexpensive as possible, while still being able to provide useful information on the in vivo behavior of the drug, to accelerate drug development [15].

In this study, two formulations based on different technologies, namely an amorphous formulation of CBD and a CBD lipid-based formulation, previously developed and having proven an increased in vivo bioavailability in piglets, were compared in terms of dissolution results in several in vitro dissolution media. An in vitro–in vivo relationship between in vitro and in vivo data has been used to confirm the choice of a dissolution medium to be used to characterize the in vivo behavior of those two different formulations.

## 2. Materials and Methods

### 2.1. Materials

CBD was purchased from THC Pharm (Frankfurt, Germany); mesoporous silica (MS) Syloid^®^ XDP 3050 and Silsol^®^ were kindly donated by W.R. Grace (Worms, Germany), and Gelucire^®^ 50/13 was kindly gifted by Gattefossé (Saint-Priest, France). FaSSGF/FaSSIF/FeSSIf-V2 powder was purchased from Biorelevant^®^ (London, UK). CO_2_ was supplied from Air Liquide (Liège, Belgium). Acetonitrile was HPLC-grade and purchased from J.T. Baker (Gliwice, Poland); Milli-Q Water was produced with a Purist UV (RePhile Bioscience). Hydrochloride acid 37% was purchased from Merck (Darmstadt, Germany). Sodium lauryl sulfate (SLS), Na_2_HPO_4_ and NaH_2_PO_4_.2H_2_O were provided by Sigma-Aldrich (Saint-Louis, MO, USA), VWR (Leuven, Belgium) and Merck (Darmstadt, Germany), respectively.

### 2.2. Production and Characterization of CBD Formulations

#### 2.2.1. Amorphous Formulation

The amorphous formulation used in this study has been developed and published by the current team [10]. It consists of mesoporous silica Silsol^®^ (pore size: 6.6 nm) impregnated by CBD (60:40) in subcritical CO_2_ conditions. A high-pressure cell (TOP Industrie, Vaux-le-Pénil, France) was filled with an appropriate mass of MS and CBD. Once closed, the cell was immerged in a water bath set at 50 °C and filled with CO_2_ at the pressure of 60 bars, with magnetic stirring set at 200 rpm. The system was finally depressurized after one h.

#### 2.2.2. Lipid-Based Formulation

The lipid-based formulation was also developed by the current team [16]. It consists of an eutectic mixture of CBD and Gelucire^®^ 50/13 incorporated in mesoporous silica XDP (pore size: 25 nm) in proportions 20:40:40 (CBD:Gelucire:XDP). CBD and Gelucire were melted in a water bath set at 70 °C. Once both components were melted and homogeneously mixed, MS XDP was manually added to obtain a free-flowing powder.

#### 2.2.3. Solubility of Pure API in the Several Media

The solubility of pure CBD in different media was tested by the shake-flask method [17]. An excess of CBD was dispersed in 10 mL of medium placed in an opaque flask and shaken for 24 h at 37 °C at 100 rpm. Samples were then filtered through a PTFE (0.45 µm) filter and diluted prior to HPLC-UV to assess the CBD maximum solubility in each used medium. No drug adsorption on the filter was observed.

#### 2.2.4. HPLC-UV

Drug content and dissolution tests were analyzed by a previously validated analytical method [10] that consisted of a Zorbax^®^ C18 300 SB analytical column with particles of 3.5 μm (150 mm × 4.6 mm ID). The mobile phase was composed of a mixture of water and acetonitrile (38/62% *v*/*v*). The flow rate was set at 1.0 mL/min, the column temperature was kept constant at 30 °C and the detection wavelength was 240 nm. The selectivity of the method has been proved by using THC and cannabielsoin.

#### 2.2.5. In Vitro Dissolution Tests

Dissolution tests were performed in a paddle (USP II) apparatus with a rotation speed set at 100 rpm to study the release of CBD from the two different formulations in different dissolution media of a volume of 500 mL. The choice of the apparatus and rpm was based on earlier results [10]. The different media were selected based on solubility results and consisted of HCl 0.1 N with and without 0.5% SLS (sodium laurylsulfate), phosphate buffer pH 6.8 with and without 0.5% SLS, and FaSSGF/FaSSIF/FeSSIF-V2.

An equivalent of 65 mg of CBD (non-sink condition, for all media) in 00-sized capsules and 10 mg of CBD (sink condition, FaSSIF medium) were used in 500 mL of medium set at 37 °C ± 1. The sink condition was obtained by reducing the CBD dose. Japanese basket sinkers were used to ensure appropriate immersion of the capsules. Each test was conducted for four hours (withdrawals at 5, 15, 30, 45, 60, 90, 120, 180 and 240 min) in triplicate. Each withdrawal was filtered through a 0.45 µm PTFE filter prior to HPLC-UV dosage.

The dissolution curves were modeled using the Weibull equation (Equation (1)).

(1) $$F_{\inf}*{\left(1-e^{\left(\frac{t}{\mathrm{MDT}}\right)}\right)}^{b}=\%\;\mathrm{dissolved}$$

where F_inf_ is the maximum % dissolved drug in dissolution medium, t is the time, MDT is the mean dissolution time and b is the slope factor.

#### 2.2.6. In Vivo Pharmacokinetic Data

The pharmacokinetic data used in this study were obtained from a previous study conducted by the current team [11]. Briefly, amorphous and lipid-based formulations (which contained an equivalent of 2.5 mg/kg of CBD) were administered orally to fasted piglets (n = 5 mean weight of 15 kg) and plasma collected at times zero, 1 h, 2 h, 2.5 h, 3 h, 3.5 h, 4 h, 4.5 h, 5 h, 5.5 h, 6 h, 7 h, 8 h, 12 h and 24 h was analyzed by a validated UHPLC–MS/MS method [18]. The mean pharmacokinetic parameters C_max_ and AUC_last_ of each formulation were used. These results are fully discussed elsewhere, and the curves are presented in the supplementary materials (Figure S1).

#### 2.2.7. In Vitro–In Vivo Relationship

In vitro data were compared to in vivo data using a point-to-point relationship (in vitro–in vivo correlation level A). Pharmacokinetic modeling and prediction were performed using Phoenix^TM^ WinNonlin 8.4 (Certara, Princeton, NJ, USA). In vivo absorption profiles of CBD from both formulations were obtained by using a deconvolution method using UIR values obtained from a previous study of intravenously injected CBD solutions in another group of piglets [18]. The results were expressed in cumulative input and fraction input. Cumulative input provided a profile ending at absolute bioavailability (F_max_) per animal in the function of the formulation and is expressed in percentage. Fraction input or absorbed fraction expressed relative to F_max_ × D provided a profile ending at 1 (fraction) or 100% (percentage), representing more the rate of absorption as independent from the extent (F_max_). The relation between in vitro and in vivo data was based on time scaling approach where the in vitro time to reach a target % input was related to the in vivo time to reach the same % input by using an inverse release function (IRF) equation (Equation (2)) [19]. It provides the equivalent in vitro dissolution time as a function of the in vivo absorbed fraction of the drug in a considered medium and is expressed as time scaling.

(2) $$t\;\mathrm{eq}.\;\mathrm{in}\;\mathrm{vitro}=-\mathrm{Ln}{\left(\frac{-{\mathrm{FD}}_{\mathrm{abs}}\%}{F_{\inf}}+1\right)}^{\frac{1}{b}}*\mathrm{MDT}$$

where FD_abs_% is the percentage of the absorbed fraction of the drug.

The averages of predicted values have been used and compared to the mean observed values. Model predictability was estimated internally for the pharmacokinetic parameters AUC and C_max_. Media with the lowest average percentage prediction error (% PE) were selected as promising.

## 3. Results

### 3.1. CBD Maximum Solubility

The pure solubility of CBD in several media is gathered in Table 1. The values are highly influenced by the pH and the presence of surfactants. Degradation of pure CBD in acidic media was noticed with a higher presence of degradation peaks in chromatograms linked to non-biorelevant media versus FaSSGF. No degradation was noticed in any neutral medium. The solubility of CBD is enhanced by the presence of surfactants such as the non-biorelevant SLS or as biorelevant lecithin and sodium taurocholate, contained in biorelevant media (FaSSGF, FaSSIF and FeSSIF). The lower lecithin and sodium taurocholate concentrations (0.02 and 0.08 mM, respectively) in FaSSGF compared to those in FaSSIF (0.75 and 3 mM) and FeSSIF (3.75 and 15 mM), coupled with detected degradation, explain the unmeasured value in FaSSGF medium. Surprisingly, no difference between FaSSIF and FeSSIF was observed, although there are five more surfactant concentrations in FeSSIF.

### 3.2. In Vitro Characterization

**Figure 1:**
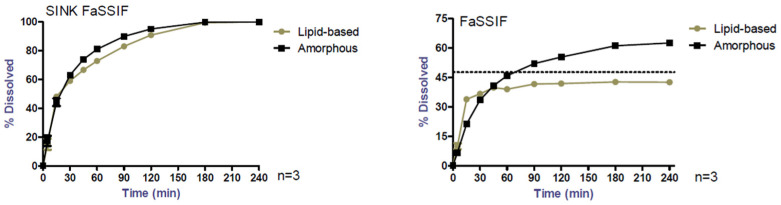
Release profiles of CBD in FaSSIF medium, under sink and non-sink conditions; please note the different scale.

The CBD content in all formulations used in this study was between 95 and 105%. Dissolution tests may be performed under sink or non-sink conditions depending on the purpose of the test. The release rates of both formulations in FaSSIF medium under sink conditions are presented with the release rates under non-sink conditions (Figure 1). Under sink conditions, both amorphous and lipid-based formulations enabled a fast and complete CBD release [11]. By using non-sink conditions, the two formulations showed different dissolution behavior with a burst release followed by rapid equilibrium under the saturation concentration for the lipid-based formulation in contrast with the amorphous formulation.

**Figure 2:**
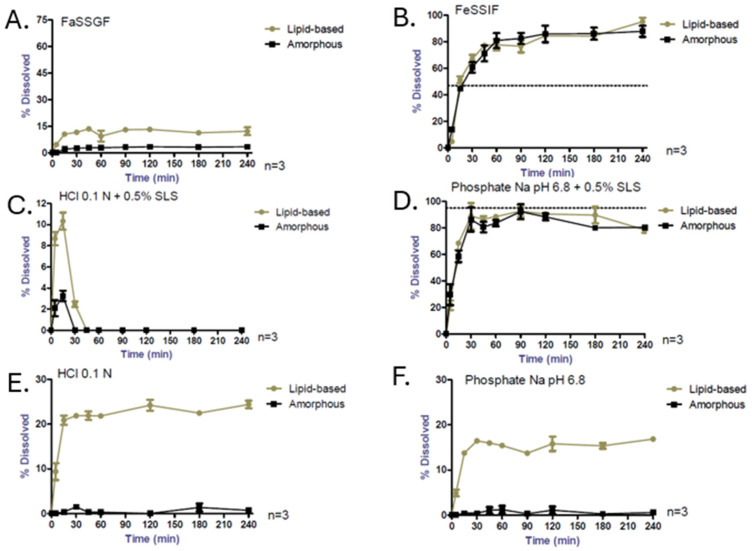
Non-sink dissolution tests of lipid-based and amorphous formulations (n = 3) in FaSSGF (**A**), FeSSIF (**B**), HCl 0.1N + 0.5% SLS (**C**), phosphate Na pH 6.8 + 0.5% SLS (**D**), HCl 0.1N (**E**) and phosphate Na pH 6.8 (**F**). Dashed lines represent the CBD’s maximum solubility. Please note the scale differences.

The dissolution results of each formulation in the other media performed under non-sink conditions are presented in Figure 2. It shows that the release rates are or are not similar in function to the chosen medium. By using HCl 0.1 N + 0.5% SLS, FaSSGF, phosphate buffer pH 6.8 + 0.5% SLS and FeSSIF-V2, no difference greater than 10% between amorphous and lipid-based formulations could be drawn contrary to the use of HCl 0.1 N, phosphate buffer pH 6.8 and FaSSIF. However, significant CBD degradation was observed by using HCl 0.1 N + 0.5% SLS for both formulations, leading to misinterpretation of results. Finally, only HCl 0.1 N, phosphate buffer pH 6.8 and FaSSIF media under non-sink conditions were able to differentiate the two studied formulations. Concerning the simplest media HCl 0.1 N and phosphate buffer pH 6.8 without any surfactant, no CBD solubilization occurred with the amorphous formulation, while the lipid-based formulation, which contains surfactant properties, enabled CBD release. Only neutral media supplemented by surfactant (SLS or lecithin/sodium taurocholate) showed high solubilization properties with the amorphous formulation. Lipid-based formulation enabled, in contrast, CBD solubilization in each tested medium whether surfactants were present or not, with a maximum of concentration reached after 30 min. A decrease in CBD concentration over time was observed for both formulations in phosphate buffer with SLS.

No degradation was noticed with the two formulations in any neutral medium. Concerning acid media, no degradation was detected by using amorphous or lipid-based formulations in HCl 0.1 N and in FaSSGF, while high degradation was observed in HCl 0.1N + 0.5% SLS for both formulations. Only FaSSIF sink, phosphate buffer pH 6.8 + 0.5% SLS and FeSSIF enabled us to reach complete dissolution.

### 3.3. In Vivo Results

**Figure 3:**
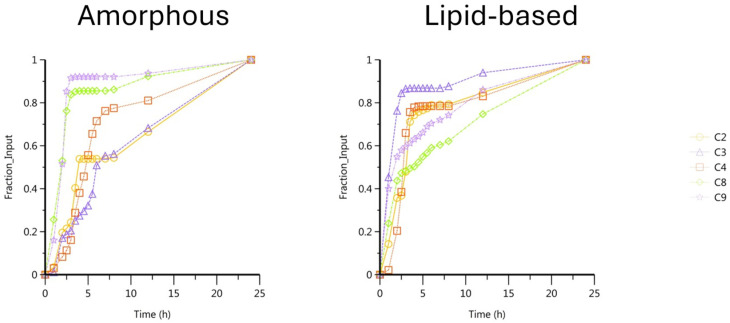
CBD fraction absorption per animal, from amorphous and lipid-based formulations.

The plasmatic concentrations used in this work were obtained from a previous study [11]. Deconvolution processes were performed by using UIR values obtained from another group of piglets [18] and enabled us to estimate the CBD absorption. The fraction absorption represents the kinetic of the total amount absorbed up to F_max_ × dose (FD) and all absorption profiles, per animal and per medium, are plotted in Figure 3 and finished at 1 (100%).

The cumulative absorption represents the absolute bioavailability, and the F_max_ values are shown in Table 2.

The amorphous formulation presented a mean absolute bioavailability of 24% and the lipid-based formulation provided a value of 14%. Both cumulative (Table 2) and fraction absorption values (Figure 3) showed a high variability between the animals. In particular, CBD showed a variable bioavailability from the amorphous formulation depending on the piglet considered, which could be linked to either poor solubilization or a variable first-pass metabolism.

### 3.4. Finding the Best In Vitro Method

For calculating correlations based on in vivo absorption reaching values up to one (100%), the sink condition may be preferred, as in vitro and in vivo absorption are all expressed up to 100%. Indeed, non-sink dissolution tests could only be related to in vivo data if the absorption expressed as F_max_ is used, but we assume that the poor bioavailability is only due to poor solubility and not to other phenomena such as the variable first pass effect. This could lead to possible misinterpretation as the results could be driven by in vivo quantities finally reaching the blood and not the rate. In the present cases, the dissolution tests in acidic media (FaSSGF, HCl and HCl + 0.5% SLS) and in phosphate buffer pH 6.8 without any surfactant were excluded since the maximum of dissolution in each of these media was lower than the maximum of in vivo absorption.

The in vitro–in vivo relations were therefore focused on the dissolution tests performed in FaSSIF (sink and non-sink conditions), in FeSSIF (non-sink condition) and in phosphate buffer added with 0.5% SLS (non-sink condition) due to the sufficient CBD release.

**Figure 4:**
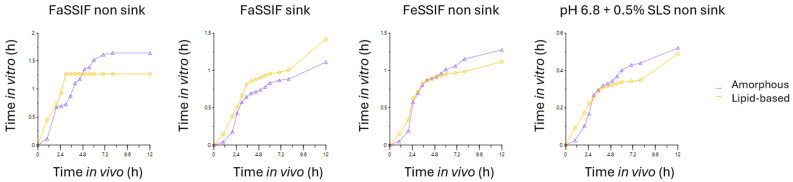
Relations between in vitro and in vivo times depending on the medium.

The relation between the in vitro and in vivo results has been compared by a time scaling approach. Dissolution curves were modeled by the Weibull equation and reached a suitable correlation in all cases with r^2^ > 0.99. The deconvolutions of the pharmacokinetic data were obtained by using the UIR obtained by injection of intravenous CBD in piglets from another study. The use of IV parameters from another study assumes a constant clearance between animals of both studies. These absorption profiles were then correlated by using the inverse release function (IRF) with the different dissolution tests. Figure 4 shows per medium the relation between the in vivo and in vitro times to have the same percentage of input, averaged per formulation.

These results show that the use of FaSSIF medium under non-sink conditions is not optimal and leads to differences between formulations. Different shapes between the two formulations are spotted with a plateau formed with the lipid-based system after a fast initial release. The use of phosphate buffer pH 6.8 added with SLS also provided shapes closer to FeSSIF. FaSSIF sink and FeSSIF media enabled better similarity between the shape of curves.

The calculated mean time scaling was then used to predict absorption profiles from in vitro data. Predicted absorption inputs have been calculated up to respective F_max_ values (Table 2). Indeed, absorption input cannot exceed the in vivo maximum absorption. Predicted absorption profiles were used as an input function to predict pharmacokinetic curves via convolution. Because the studies which featured the intravenous data providing the UIR values were not carried out on the same individuals as those presented in this study, the variability of each animal in terms of clearance was considered by adapting the predicted absorption input with the F_max_ related to each piglet (Table 2). The mean calculated values of predicted C_max_ and AUC were confronted with observed values and the prediction errors (PE%) are shown in Table 3.

The prediction of both AUC and C_max_ was not conclusive by using FaSSIF medium under non-sink conditions. FaSSIF medium under sink conditions, FeSSIF medium and phosphate buffer pH 6.8 (+0.5% SLS) medium under non-sink conditions enabled appropriate AUC prediction for both formulations (Table 3). Overall, FaSSIF sink and FeSSIF composition were the media enabling correct prediction of C_max_. Those different predictions may be linked to the concentrations of fatty surfactants related to the quantity of CBD in the different media, expressed in a ratio and shown in Table 4. The higher the ratio, the better the prediction.

**Figure 5:**
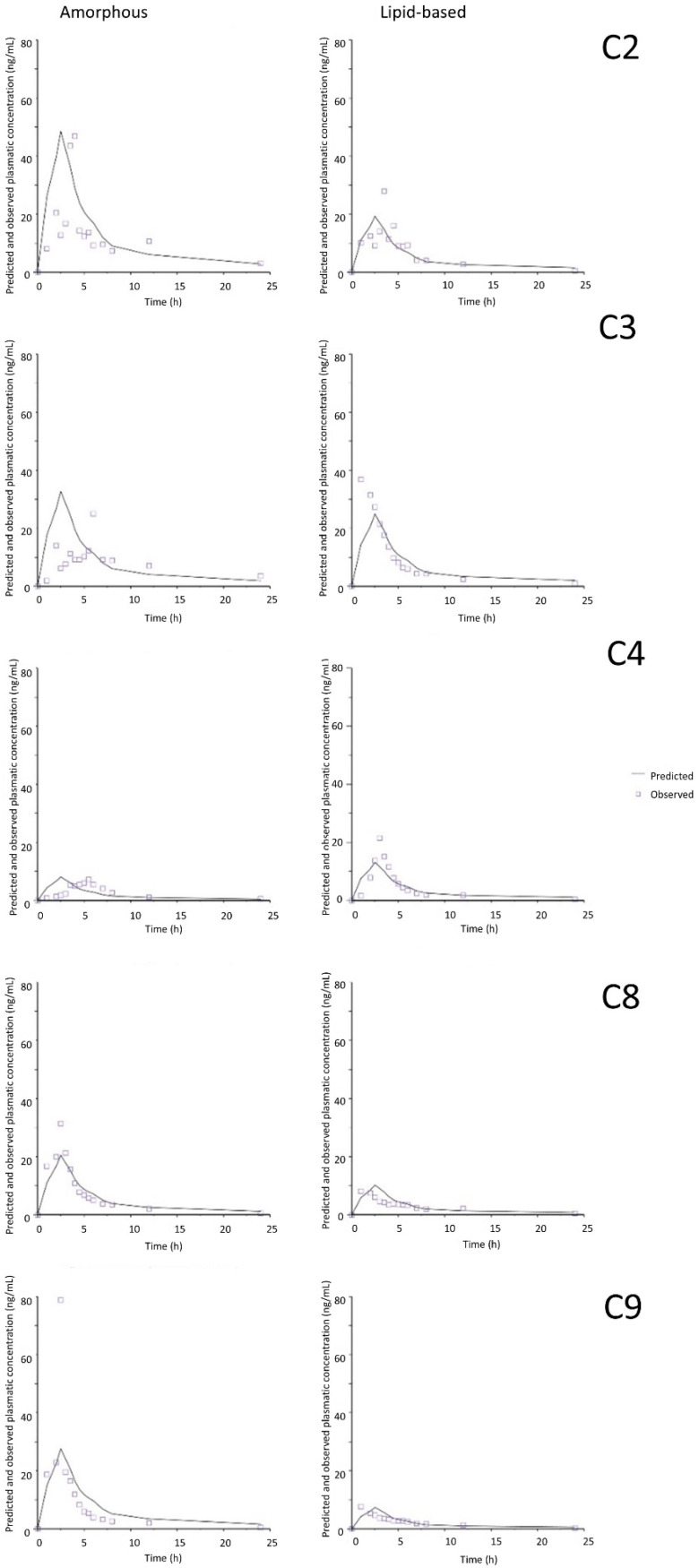
Plasma concentration–time curves of predicted and observed values in FaSSIF sink medium and by using common time scaling. C2–C4, C8 and C9 correspond to the five different animals.

**Figure 6:**
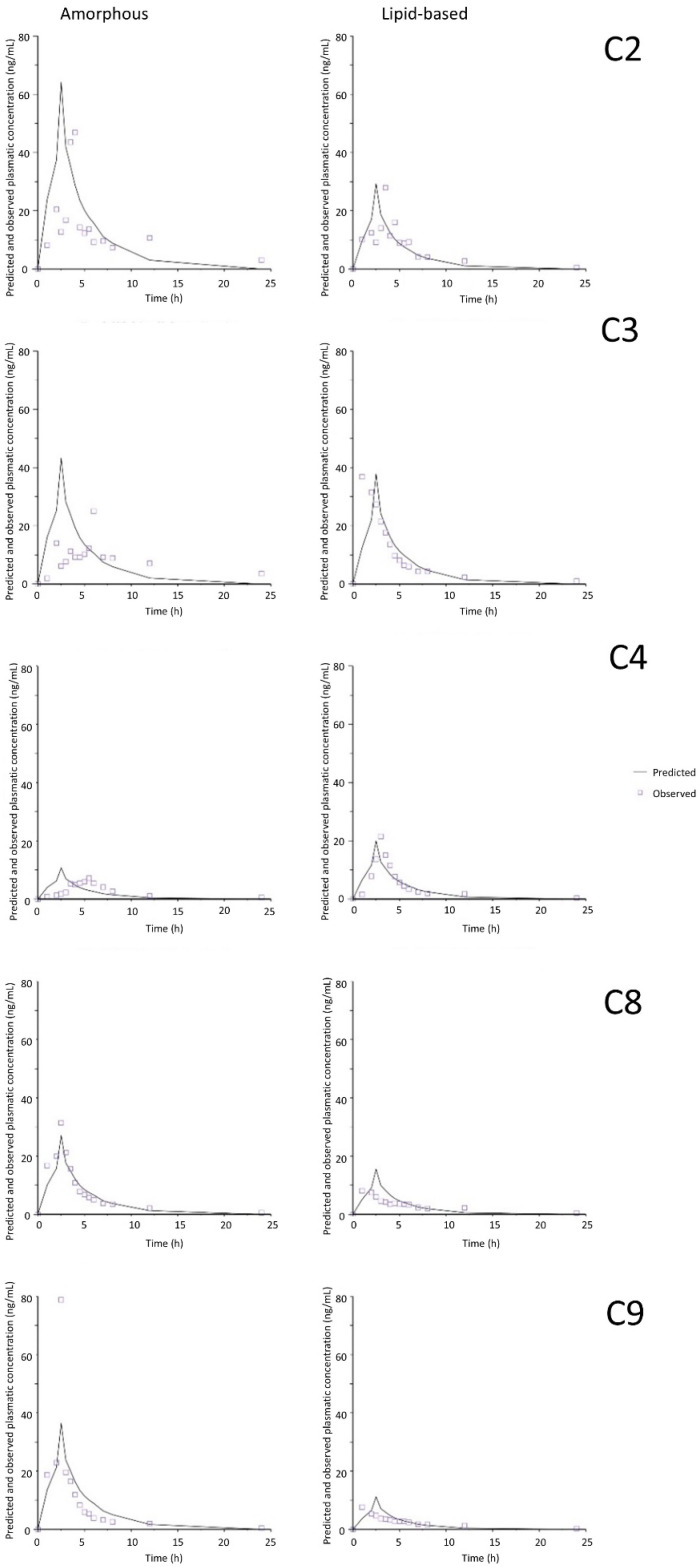
Plasma concentration–time curves of predicted and observed values in FeSSIF medium and by using common time scaling. C2–C4, C8 and C9 correspond to the five different animals.

Figure 5 and Figure 6 show the observed plasma concentrations versus time values and their respective predicted values for each piglet in the two media containing the highest ratio of surfactants to CBD. The relatively good superposition of the observed and predicted curves is explained by the low prediction errors.

## 4. Discussion

The use of in vitro dissolution tests is very useful to characterize, in an easy manner, formulations developed to improve the solubility and the bioavailability of pharmaceutical compounds. The first aim of this work was to study the impact of the dissolution medium on the discrimination of two different CBD formulations. The importance of the choice of several dissolution parameters on the dissolution results, and thus of the interpretation of the results, has been highlighted. While the sink condition is generally recommended to appreciate the release rate of a drug from its formulation, no information about the capacity of producing a supersaturated state is provided. In the context of the development of formulations with enhanced aqueous solubility, non-sink conditions may be useful since the results could indicate an increase in kinetic solubility as well as precipitation behavior [20]. Non-sink conditions were therefore the initial primary choice in this work, as the two developed and tested formulations were optimized to achieve supersaturation, maintained for the maximum time. However, these conditions only reflect the initial solubilization, but in vivo, the conditions could be largely different, and absorption could occur, rapidly decreasing the local concentrations. In this respect, non-sink conditions could only reflect the initial in vivo dissolution but no further phenomena such as the role of absorption in the decrease in local concentrations especially for the class II drug. This in vivo behavior may decrease the interest in non-sink conditions as these conditions may not be present in vivo as they are in vitro.

CBD release has been shown to be highly influenced by the medium composition. In fact, CBD has a pKa value around 9 and its solubilization is therefore not influenced by physiological pH. The major parameter that influences its solubility is its high lipophilicity (logP = 6.3) [21]. Indeed, CBD has poor interaction with water and requires surfactants, such as SLS, or physiological surfactants and fat (lecithin and sodium taurocholate present in digestive fluids and FaSSGF/FaSSIF/FeSSIF media) to improve its solubility. The biorelevant media might better mimic the possibilities of dissolution in vivo even if in vivo absorption could be a major factor to be considered to decrease local quantities of drug in the lumen.

Drug absorption combines two aspects, namely the dissolution rate K_d_ and the permeability rate K_p_. As a BCS II drug, CBD should not be limited by its permeability but by its solubilization. In vivo, the dissolution rate of CBD will be greater if its solubility is increased. K_d_ is therefore the limiting step when CBD absorption is considered. The observed low oral bioavailability is not linked with only a low and limited solubilization in vivo but also by the impact of first-pass metabolism which occurs after the permeation of the drug and consequently does not reflect in vivo dissolution [11]. It implies that in vitro non-sink conditions, which reflect a low and incomplete dissolution and which could be reflecting, in some cases, the in vivo F_max_, might not reflect the real in vivo dissolution.

Linking the in vivo prediction to in vitro dissolution tests is, in this sense, challenging. On the one hand, we can only determine the dissolution rate using sink conditions. Non-sink conditions give information on the CBD maximum solubility in a fixed volume (which might not represent in vivo conditions), but the release rates are not pertinent, since they are influenced by the limited solubility and ignore any absorption occurring in vivo. More complex systems, such as the use of biphasic dissolution tests, are sometimes used to cover both dissolution and supersaturation events. Indeed, biphasic tests consist of an aqueous dissolution medium, in which the formulation is immersed, placed under an organic medium, such as octanol. Even more complex systems, such as slightly modified USP apparatus type II (a second paddle is added for the organic phase agitation) combined with type IV apparatus, have been developed [22]. The aim of such complex systems is to represent the absorption process, because of the diffusion of the solubilized drug within the organic phase. The drug concentration in the aqueous medium may not influence the overall dissolution, since any excess will transfer to the organic phase, partially limiting the non-dissolution that occurs in non-sink conditions [23]. It should, however, be mentioned that biphasic tests are more complicated to implement compared to monophasic systems and require customized equipment [14]. To enable an easy-to-implement dissolution test, we have therefore focused our work on monophasic dissolution tests.

Despite this challenge, combining in vivo data to in vitro data helps to avoid mischaracterizing formulations in development and is a very useful tool for selecting the right in vitro model and then enable possible in vivo behavior of formulations.

The second aim of this study was to select a dissolution medium which enables us to predict the in vivo fate of CBD from qualitatively different formulations. However, a large number of judicious choices must be made to build a prediction model.

Since the percentage of predicted input (predicted absorption profiles) is based on the equation of dissolution, starting from an input which goes from 0 to 100% in theory and from time scaling calculated on fraction input, calculated values may be corrected by the maximum of absorption F_max_, especially in the present case as the UIR model belongs to another study with different animals and the convolution assumes a constant clearance.

As CBD, from the amorphous formulation, was very poorly soluble in acidic media such as HCl 0.1 N (±0.5% SLS) and FaSSGF, but also in phosphate buffer pH 6.8 without SLS, these media were not included in the prediction models. Intuitively, it is already clear that a correlation between an in vivo curve showing sufficient AUC and an in vitro curve showing little or no % dissolved is unlikely. The in vivo contact of the formulations with an acidic environment may have only limited impact, as indicated by dissolution tests performed in FaSSGF as no or little release of CBD coupled with no degradation has been observed. Our hypothesis is that the gastric residence of the formulations in fasted piglets delayed the in vivo CBD release without having a negative impact. If CBD may be converted into other cannabinoids, such as THC under a non-physiological acidic medium, the presence of complex biological components such as lecithin or sodium taurocholate may have a role in the protection of CBD degradation. This topic has been reviewed by Golombek et al., where the authors concluded that CBD is converted into THC upon treatment with strong acids, but this conversion is not observed in the majority of animal studies [24]. This is another example of the challenge linked to the high differences between the in vitro and in vivo situations, and further studies have to be conducted to investigate the reason for the non-conversion of CBD in in vivo conditions. In the context of our work, which aims to select a biorelevant dissolution test which is easy to implement, acidic media are clearly not an option. The neutral media that contain surfactants used in this study, except FaSSIF medium under non-sink conditions, were the most appropriate to predict the in vivo parameters of both amorphous and lipid-based formulations. FaSSIF sink, FeSSIF, and pH 6.8 + 0.5% SLS enabled a dissolution greater than 80%. C_max_ prediction was more difficult as different populations of T_max_ exist and are highly dependent on animal individuals. FeSSIF and FaSSIF sink media, being the media with the highest surfactant capacity and containing fat (taurocholate sodium and lecithin), were the optimal choice since they enable better C_max_ and AUC prediction for both amorphous and lipid-based formulations. FeSSIF, which most closely represents the fed state, correlates best with the in vivo results in the present study even if the drug was administered in a fasted state. This shows the interaction of the two formulations with the composition of the dissolution media in terms of surfactant quantity. This could also explain the fact that FaSSIF in sink conditions provides interesting results, even if it is less promising in terms of C_max_ values than FeSSIF. This is also confirmed by pH 6.8 added with 0.5% of SLS medium, which is not able to accurately predict the C_max_ of amorphous formulations even if surfactants are present. An absence of or low lecithin quantities impaired the accurate prediction of the C_max_ of amorphous formulations. The most accurate predictions occur with FeSSIF (higher lecithin quantities) followed by FaSSIF sink (lower lecithin quantities) and then by pH 6.8 + 0.5% SLS, which does not contain any fat. The requirement for fatty compounds, such as lecithin and sodium taurocholate, has already been demonstrated to enable the formation of mixed micelles that improve the reflection of the upper part of the gastrointestinal tract, and the addition of fatty materials like Capmul^®^ MCM in biorelevant media or in phosphate buffer pH 6.8 added with SLS may provide better pharmacokinetic predictions [25].

Along these lines, this study demonstrates the capabilities of several dissolution media, especially FeSSIF, to characterize optimized formulations of CBD. These results possibly indicate an in vivo solubilization mechanism related to the formation of micelles, which is more reflected by media with a high quantity of surfactants and fat.

Finally, this study shows how important the choice of dissolution parameters is to consider and link with the role of this test. In fact, the FeSSIF medium under non-sink conditions provided the best predictions of pharmacokinetic parameters from two formulations with different solubilization mechanisms.

## 5. Conclusions

In vitro dissolution tests are challenging tools that might provide relevant in vivo data. The selection of the parameters, such as sink or non-sink conditions, dissolution medium and settings, must be based on their relevance versus the in vivo results. In this study, two CBD formulations (lipid-based and amorphous) were compared to select a discriminant dissolution test. Since CBD is highly lipophilic, tests that are expected to mimic in vivo situations must have surfactant capacity and micelle formation capacities linking with the presence of fats. In vitro–in vivo correlation has been investigated and the suitability of neutral media such as FaSSIF or phosphate buffer pH 6.8 added with SLS has been demonstrated to predict the in vivo behavior of both formulations in terms of the AUC, but it still lacks precision in terms of C_max_ prediction. FeSSIF medium under non-sink conditions and, to a lesser extent, FaSSIF in sink conditions enabled AUC and C_max_ prediction for both formulations.

## Figures and Tables

**Table 1:** Maximum pure CBD solubility in several media. LOQ = limit of quantification.

Medium	CBD Solubility (mg/mL, 24 h, 37 °C) ± SD	Degradation
HCl 0.1N	<LOQ	Yes (++)
HCl 0.1N + 0.5% SLS	0.00104 ± 0.00017	Yes (++)
Phosphate buffer pH 6.8	0.00087 ± 0.00099	No
Phosphate buffer pH 6.8 + 0.5% SLS *	0.06471 ± 0.00164	No
FaSSGF	<LOQ	Yes (+)
FaSSIF *	0.06217 ± 0.00270	No
FeSSIF *	0.06270 ± 0.00254	No

* The volume needed for FaSSIF, for FeSSIF and for the pH 6.8 + SLS is more than 3 L to be in sink conditions for a 65 mg dose. Degradation is considered as strong if “++” and as mild if “+”.

**Table 2:** F_max_ of CBD from the two formulations.

Animal	Amorphous	Lipid-Based
C2	43%	18%
C3	29%	24%
C4	7%	13%
C8	18%	10%
C9	25%	7%
Mean (CV)	24% (54%)	14% (47%)

**Table 3:** Prediction errors by using time scaling and mean NCA (n = 5).

	Amorphous		Lipid-Based	
Medium	C_max_ Prediction	AUC Prediction	C_max_ Prediction	AUC Prediction
FaSSIF	49.28	42.42	40.94	50.27
FaSSIF sink	19.04	7.67	18.99	2.98
FeSSIF	7.05	8.55	22.62	10.66
pH 6.8 SLS	37.41	12.91	10.41	6.51

**Table 4:** Surfactant/CBD ratio depending on the dissolution parameters.

Ratio Surfactant/CBD	FaSSIF (Lecithine + Sodium Taurocholate)	FeSSIF (Lecithine + Sodium Taurocholate)	FaSSIF Sink (Lecithine + Sodium Taurocholate)	pH 6.8 (SLS)
Amorphous	16.78	83.85	109	38.46
Lipid-based	18.77	85.85	111	40.46

## Data Availability

The data presented in this work are available on demand.

## Supplementary Materials

The following supporting information can be downloaded at: https://www.mdpi.com/article/10.3390/pharmaceutics17010079/s1, Figure S1 Mean CBD plasmatic curves from the amorphous and lipid-based formulations, compared with crystalline CBD (n = 5).
