# Supplementary material for: Selection of In Vivo Relevant Dissolution Test Parameters for the Development of Cannabidiol Formulations with Enhanced Oral Bioavailability
Source: Pharmaceutics. 2025 Jan 9;17(1):79. doi: 10.3390/pharmaceutics17010079 (PMC11769287; doi:10.3390/pharmaceutics17010079)
Supplement: Supplementary file 1 [file pharmaceutics-17-00079-s001.zip › pharmaceutics-3392364-supplementary.pdf]

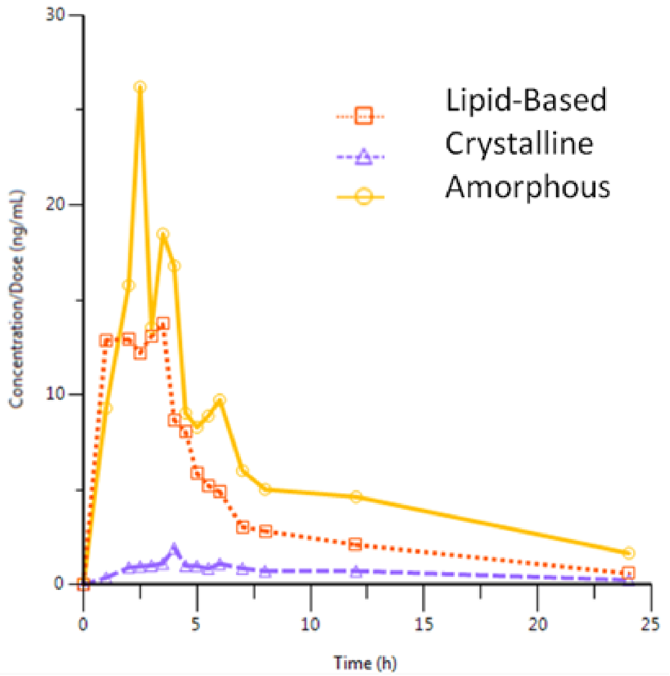

**Figure S1.** Mean CBD plasmatic curves from the amorphous and lipid-based formulations, compared with crystalline CBD (n=5).
